# Therapeutic targeting potential of the protein lysine and arginine methyltransferases to reverse cancer chemoresistance

**DOI:** 10.3389/fmolb.2024.1455415

**Published:** 2024-12-05

**Authors:** Isaac Micallef, Kimberly Fenech, Byron Baron

**Affiliations:** ^1^ Centre for Molecular Medicine and Biobanking, University of Malta, Msida, Malta; ^2^ Department of Tumor Genetics and Biology, Graduate School of Medical Sciences, Faculty of Life Sciences, Kumamoto University, Kumamoto, Japan

**Keywords:** chemoresistance, methylproteomics, protein methylation, protein methyltransferases (PMTs), PMT inhibitors

## Abstract

Cancer treatments have continued to improve tremendously over the past decade, but therapy resistance is still a common, major factor encountered by patients diagnosed with cancer. Chemoresistance arises due to various circumstances and among these causes, increasing evidence has shown that enzymes referred to as protein methyltransferases (PMTs) play a significant role in the development of chemoresistance in various cancers. These enzymes are responsible for the methylation of different amino acids, particularly lysine and arginine, via protein lysine methyltransferases (PKMTs) and protein arginine methyltransferases (PRMTs), respectively. Various PMTs have been identified to be dysregulated in the development of cancer and chemoresistance. Nonetheless, the functional role of these PMTs in the development of chemoresistance is poorly characterised. This advocates the need for innovative approaches and technologies suitable for better characterisation of these PMTs and their potential clinical inhibitors. In the case of a handful of PMTs, inhibitory small molecules which can function as anticancer drugs have been developed and have also entered clinical trials. Considering all this, PMTs have become a promising and valuable target in cancer chemoresistance related research. This review will give a small introduction on the different PKMTs and PRMTs families which are dysregulated in different cancers and the known proteins targeted by the respective enzymes. The focus will then shift towards PMTs known to be involved in chemoresistance development and the inhibitors developed against these, together with their mode of action. Lastly, the current obstacles and future perspectives of PMT inhibitors in cancer chemoresistance will be discussed.

## 1 Introduction

Chemotherapy is one of the main modes of cancer treatments, however its efficacy is limited by chemoresistance. Chemoresistance is the ability of cancer cells to survive in the presence of commonly used chemotherapeutics, ultimately increasing the morbidity and mortality of the cancer (Yeldag et al., 2018). For this reason, drug resistance is a key issue that cancer research seeks to understand and address (Yeldag et al., 2018).

Recently, several studies have demonstrated that post translational modifications (PTMs), particularly protein methylation can play a critical role in chemoresistance. PTMs are chemical modifications on certain amino acid residues, employed by cells to alter the stability, interactions, activity, distribution and conformation of various proteins (Zhu et al., 2022). Over 200 different types of PTMs have been identified, which alter cellular metabolism, signalling cascades, gene expression, DNA repair and cell cycle control (Duan and Walther, 2015). Protein methylation long recognised for its role in transcription regulation through its addition on histones, has also been identified on non-histone proteins, such as ribosomal proteins, transcription factors, structural complexes and enzymes (Dai et al., 2021; Levy, 2019). Protein methylation is added by ‘writers’ known as histone or protein methyltransferases (HMTs or PMTs), while ‘erasers’ referred to as histone or protein demethylases (HDMs or PDMs) remove methyl groups from amino acid residues (Kaniskan et al., 2018).

The addition and removal of methyl groups from proteins, together with the enzymes responsible for these processes, are known to be dysregulated in cancer. However, the relationship between protein methylation, the responsible PMTs and cancer chemoresistance remains largely unexplored. Furthermore, numerous groups have been investigating the potential of inhibiting methylation using small molecules as a therapeutic approach to counter chemoresistance.

This review will introduce the main PMT families known to be dysregulated in cancer and implicated in chemoresistance. The focus will then shift onto the available small molecule inhibitors, their mode of action and enzyme specificity. Finally, the challenges of applying these molecules in a clinical setting as well as the future prospects of PMTs in cancer chemoresistance will be discussed.

## 2 Protein methylation

Protein methylation is the transfer of a CH_3_ group from the methyl donor S-adenosyl-l-methionine (AdoMet or SAM) to the amino acid residues of a protein. Most of the methylation substrates are histones, particularly Histone 3 and 4, however various non-histone methylated proteins such as tumour protein 53 (p53), heat shock proteins 70 or 90 (HSP70 or HSP90), Nuclear factor kappa B (NF-κB), Retinoblastoma 1 (RB1), among other have been identified over the past few years (Tables 1, 2) (Hamamoto et al., 2015; Wei et al., 2014).

**TABLE 1:** Histone Methylation markers contributing to chemoresistance development.

Protein Lysine Methyltransferases (PKMTs)				
Methylation Site/s	PMT/s	Treatment/Cancer	Uncovered mechanism of resistance	References
H3K9me1	*G9a or EHMT2*	Cisplatin resistant head and neck squamous cell carcinoma (HNSCC)	This study demonstrated that high G9a expression is significantly associated with poor chemotherapeutic response and disease-free survival in HNSCC patients. Moreover, G9a expression and enzymatic activity was elevated in cisplatin-resistant HNSCC cells. Genetically or pharmacologically inhibiting G9a re-sensitised resistant cells to cisplatin treatment. Mechanistic investigations uncovered that G9a plays a role in the transcriptional activation of the glutamate-cysteine ligase catalytic subunit (GCLC) by regulating the level of H3K9me1 at the *GCLC* promoter. This activation results in increased cellular glutathione (GSH) levels, contributing to drug resistance	Liu et al. (2017)
H3K9me2	*G9a or EHMT2*	Erlotinib resistant Non-small cell lung cancer (NSCLC)	G9a exerts its influence on the PTEN promoter region by enhancing H3K9 dimethylation (H3K9me2) while simultaneously reducing H3K9 acetylation (H3K9ac). This epigenetic modification resulted in the transcriptional suppression of PTEN. Consequently, the downregulation of PTEN led to the activation of the AKT signalling pathway. The heightened AKT signalling, in turn, contributed to the development of erlotinib resistance. Combination treatment with the EHMT2 inhibitor and erlotinib resulted in enhanced antitumor effects in a preclinical EGFR-TKI-resistance model	Wang et al. (2018a)
H3K9me3	*SUV39H1, SUV39H2, G9a/GLP*	5FU resistant colon carcinoma cells	The three PMTs, SUV39H1, SUV39H2, and G9a cooperated in catalyzing trimethylation of H3K9 on the *FAS* promoter, resulting in decreased Fas expression in metastatic human colon carcinoma cells. *FAS* was identified as a target gene of verticillin A, which is a selective inhibitor against all three PMTs. This inhibitor decreased H3K9me3 levels in the *FAS* promoter and restored Fas expression, and also exhibited great efficacy in overcoming colon carcinoma resistance to FasL-induced apoptosis. Use of this inhibitor helped overcome metastatic colon carcinoma resistance to 5FU *in vitro* and *in vivo*	Paschall et al. (2015)
H3K27me3	*EZH2*	Cisplatin, etoposide or irinotecan resistant SCLC	Multiple chemoresistant models demonstrated suppression of the Schlafen family member 11 (*SLFN11*), a factor implicated in DNA-damage repair deficiency. *In vivo* silencing of *SLFN11* was linked with marked deposition of H3K27me3 arising by EZH2, within the gene body of *SLFN11*, which lead to induced local chromatin condensation and gene silencing	Gardner et al. (2017)
		Oxaliplatin resistant colorectal cancer (CRC)	lncRNA P53 inHibiting LncRNA (PiHL) antagonised chemosensitivity through binding with Enhancer of zeste 2 (EZH2), repressing localisation of EZH2 to *HMGA2* promoter, and downregulating methylation of H3K27me3 level in *HMGA2* promoter, resulting in HMGA2 activation. HMGA2 upregulation induced by PiHL promoted PI3K/Akt phosphorylation, which lead to increased oxaliplatin resistance	Deng et al. (2021)
		Cisplatin resistant CRC	EZH2 interacted with the DNA methyltransferases (DNMT) 3a to epigenetically silence and suppress the transcription of myeloid ecotype virus insertion site 1 (MEIS1). This suppression was assisted by lncRNA ELFN1-AS1, which interacted with the two methyltransferases to locate them on the promoter of MEIS1, to increase DNA methylation and H3K27 trimethylation in the promoter region	Li et al. (2022)
		Temozolomide resistant glioma	This study presents a comprehensive understanding of how Chromobox (CBX) 2 impacts tumorigenesis, progression, prognosis and chemoresistance of glioma. It was unveiled that CBX2 recruits EZH2 to modulate the levels of trimethylation on H3K27 present on the PTEN promoter, resulting in suppression of PTEN transcription and activation of AKT/mTOR signalling pathway	Wang et al. (2024)
H3K36me2	*NSD2*	Cisplatin resistant osteosarcoma	NSD2 was upregulated in osteosarcoma tissues compared with normal tissues, as well as higher in cisplatin resistant patients than in cisplatin-sensitive ones. Its knockdown enhanced apoptosis and sensitised osteosarcoma cells to cisplatin by directly decreasing H3K36me2 levels at BCL_2_ and SOX2 gene loci	He et al. (2019)
*H3K9me2 and H3K27me3*	*G9a/EHMT2* and *EZH2*	Cisplatin or paclitaxel or erlotinib resistant NSCLC	The two PMTs, G9a/EHMT2 and EZH2 were overexpressed in resistant NSCLC. The two promoted tumour growth and mediated drug resistant in a complementary fashion. Mechanistically, it was revealed that G9a and EZH2 interact and promote the silencing of the tumour-suppressor gene *SMAD4*, via methylation of H3K9 and K27 at its promoter, which activated the RAF/ERK/c-Myc signalling pathway to mediate resistance in NSCLC.	Zhang et al. (2024b)
H4R3me2a	*PRMT1*	Cisplatin resistant ovarian cancer	PRMT1 was identified as a regulator of arginine methylation in ovarian cancer cells treated with cisplatin. It was revealed that DNA dependent protein kinase (DNA-PK) binds to and phosphorylates PRMT1 in response to cisplatin, inducing its chromatin recruitment and redirecting its enzymatic activity toward R3 of histone H4 (H4R3). On chromatin, the DNA-PK/PRMT1 axis induced senescence-associated secretory phenotype (SASP) through H4R3me2a deposition which protected from apoptosis cells confronted with sustained DNA damage. PRMT1 inhibition resensitised cells to cisplatin	Musiani et al. (2020)
H3R8me2s	*PRMT5*	5-Fluorouracil (5FU) resistant CRC	High expression of PRMT5 resulted in increased cell proliferation due to the PRMT enhancing glycolysis by transcriptionally activating Lactate dehydrogenase A (LDHA) expression through the increased occupancy of heterochromatin markers H3R8Me2s on the LDHA promoter. Inhibition of PRMT5 by tadalafil (PRMT5 inhibitor) resulted in decreased glycolysis and increased 5FU sensitivity	Shen et al. (2022)
		DOX treated CRC	This study highlights the epigenetic role of PRMT5 in regulating Dickkopf family protein 1 (DKK1), possibility through symmetrical dimethylation of H3R8. Moreover, inhibition of DKK1 or inhibition of PRMT5 with the inhibitor CMP5, combined with DOX, resulted in increased anti-tumour effects in KRAS mutant CRC cells, but not KRAS wild type cells	Abumustafa et al. (2024)

**TABLE 2:** Non-histone Methylation markers contributing to chemoresistance development.

Protein Lysine Methyltransferases (PKMTs)				
Methylation Site/s	PMT/s	Treatment/Cancer	Uncovered mechanism of resistance	References
P53 K370me	*SMYD2*	Cisplatin resistant NSCLC	Cisplatin resistant NSCLC cells exhibited increased expression of SMYD2 and p53K370me. Mechanistically, SMYD2 inhibition enhanced p53 pathway activity and induced cell apoptosis, with methylation at K370me also decreasing. The study demonstrated the involvement of SMYD2 in cisplatin resistance through regulating p53 pathway via mono-methylation of p53 at K370	Shang and Wei et al. (2019)
AKT trimethylation (lysine residue not reported)	*SETDB1*	Cetuximab resistant CRC	In KRAS mutant CRC cells, high expression of SET domain bifurcated histone lysine methyltransferase (SETDB1) resulted in trimethylation of AKT (site not reported), which promoted AKT phosphorylation, resulting in activation of PI3K-AKT pathway. SETDB1-Akt pathway increased glycolysis and increased cell proliferation, all of which contributed to cetuximab resistance. Inhibition of SETDB1 sensitized CRC to cetuximab, independent of KRAS mutation status	Hou et al. (2020)
AKT trimethylation (lysine residue not reported)		Tamoxifen resistant breast cancer	SETDB1 contributed to endocrine therapy resistance by regulating the expression of a subset of estrogen receptors (ER) and Akt targets. The ER coregulator Proline-, glutamic acid-, and leucine-rich protein 1 (PELP1) was identified as an interactor of SETDB1. Mechanistic analysis demonstrated that interaction between the latter two played a critical role in activating Akt by facilitating methylation of Akt. Additionally, SETDB1 overexpression promoted tamoxifen resistance and PELP1 was necessary for SETDB1 mediated Akt signalling which resulted in therapy resistance in ER positive breast cancer	Liu et al. (2022)
E3-ubiquitin ligase RING finger protein 113A (RNF113A) K20me3	*SMYD3*	Cisplatin resistant SCLC	SMYD3 was a crucial factor in regulating SCLC sensitivity to alkylation-based chemotherapy. This PMT methylated RNF113A, which interfered with its ability to interact with the protein phosphatase 4 (PP4), and consequently affecting RNF113A’s phosphorylation levels. The interplay between these PTMs (methylation and phosphorylation) served as a critical mechanism for activating and maintaining RNF113A’s E3 ligase activity. This activity was essential for RNF113A’s role in responding to alkylation-induced DNA damage. Inhibiting SMYD3 restored SCLC’s vulnerability to alkylating chemotherapy	Lukinovic et al. (2022)
Twist family BHLH transcription factor 1 (TWIST1) (lysine residue + methylation degree not reported)	*SETD8*	Docetaxel resistance in breast cancer	SETD8 was shown to affect chemotherapy resistance by regulating the cell cycle and positively regulate glycolysis-mediated chemotherapy resistance by inhibiting the transcription of fructose-1,6-bisphosphatase 1 (FBP1) in collaboration with TWIST1 through methylation. KMT5A inhibits the expression of FBP1 by methylating TWIST1 and weakening its promotion of FBP1 transcription	Peng et al. (2024)
Epithelial growth factor receptor (EGFR) R198me2a and R200me2a	*PRMT1*	Cetuximab resistant CRC	Study demonstrates the importance of EGFR R198/R200 methylation which is needed for regulating EGFR functionality and resistance to cetuximab treatment. Methylation of EGFR by PRMT1 enhanced its binding to EGF, promoting receptor dimerisation and downstream signal activation. Enhanced methylation of the EGFR maintained signalling activation and cellular division, even when exposed to cetuximab, a therapeutic monoclonal antibody targeting EGFR. In patients, EGFR methylation level correlated with higher recurrence rate after cetuximab treatment and reduced overall survival	Liao et al. (2015)
		Cetuximab resistant head and neck squamous cell carcinoma (HNSCC)	In this study, Snail stimulated the expression of lymphotoxin-β (LTβ), a protein belonging to the TNF superfamily, which activated PRMT1 expression. LTβ was proven to interact with the methylated EGFR (R198 and R200) arising by PRMT1. This process ultimately enhanced the ligand-binding capacity of EGFR and promoted dimer formation, contributing to drug resistance. When the interaction between EGFR and LTβ was interfered, cetuximab resistance was reversed	Hsu et al. (2017)
Glioma associated oncogene homolog 1 (Gli1) R597me2a		Gemcitabine resistant pancreatic cancer (PDAC)	This study uncovered that Gli1, a key protein in PDAC, is modified by PRMT1 at position R597. This methylation enhanced Gli1’s ability to bind to and activate its target genes (e.g., *SNAIL1, BCL2*), which in turn boosted Gli1’s transcriptional activity. Inhibition of Gli methylation resulted in decreased oncogenic functions of Gli1 and sensitised PDAC cells to gemcitabine treatment. Examination of human PDAC tissues revealed positive correlation between PRMT1 protein levels and the total Gli1 and methylated Gli1	Wang et al. (2016)
Heat shock protein 70 (HSP70) R416 me2a + R447me2a			As PDAC progresses, PRMT1 expression gradually increases. PRMT1 methylated two conserved arginine residues on HSP70, which then binds to BCL-2 mRNA through AU-rich elements (AREs) in its 3′-untranslated region (3′-UTR). This interaction stabilises BCL-2 mRNA, leading to increased BCL-2 protein expression. Consequently, cancer cells exhibit enhanced drug resistance and anti-apoptotic properties	Wang et al. (2020a)
SOX2 R43me2a		Small cell lung cancer (SCLC)	It was demonstrated that erythropoietin-producing hepatocellular A2 (EphA2) mediated PRMT1 activation promoted the expression and molecular functions of sex-determining region Y-box 2 (SOX2). PRMT1-mediated methylation was hypothesised to have intensified the expression of SOX2 by stabilising SOX2. It was concluded that EphA2/PRMT1/SOX2 signalling axis regulated cisplatin resistance in SCLC.	Liang et al. (2022)
Phosphoglycerate dehydrogenase (PHGDH) R20me2a + R54me1	*PRMT1*	Paclitaxel resistant triple negative breast cancer (TNBC)	Study identified PHGDH methylation by PRMT1 at two arginine residues. Dimethylation of R20 competed with polyubiquitination of K21 to stabilize the active enzyme, while R54 acts to stabilize the affinity for the substrate. Furthermore, methylation of these two residues was needed for supporting the enzymatic activity of PHGDH. Methylation of this enzyme accelerated the serine synthesis pathway which activated fatty acid synthesis via stabilisation of fatty acid synthase by *S*-palmitoylation in paclitaxel resistant TNBC.	Yamamoto et al. (2024)
Heterogeneous nuclear ribonucleoprotein 1 (hnRNPA1) R31me2a	*PRMT3*	Gemcitabine resistant PDAC	This study provided first evidence that PRMT3 methylated the RNA recognition motif (RRM) of hnRNPA1. Methylation of this hnRNP at R31 helped enhance its interaction with ATP-binding cassette subfamily G member 2 (ABCG2) mRNA which in turn promoted gemcitabine resistance in PDAC.	Hsu et al. (2018)
Insulin-like growth factor 2 mRNA-binding protein 1 (IGF2BP1) R452me2a		Oxaliplatin resistant hepatocellular carcinoma	Study demonstrates that PRMT3 contributes to oxaliplatin resistance through a mechanism partially reliant on methylating IGF2BP1 at the R452 position. This methylation is crucial for IGF2BP1’s role in stabilizing HEG1 mRNA, an important component of the PRMT3-IGF2BP1 pathway. Additionally, elevated PRMT3 levels may indicate oxaliplatin resistance in hepatocellular carcinoma (HCC) patients. Overall, PRMT3-IGF2BP1-HEG1 pathway serves as a key regulator in oxaliplatin resistance, presenting potential therapeutic targets	Shi et al. (2023)
SMAD7 methylation (arginine site and methylation degree not reported)	*PRMT4 or CARM1*	Small cell lung cancer (SCLC)	CARM1 regulated methylation of SMAD7, which lead to activation of the TGF-β/Smad pathway, resulting in cisplatin resistance. This phenomena was proven to be regulated/reversed by ESRP1, which could inhibit TGF-β/Smad signalling pathway by reducing the content of CARM1, resulting in enhanced chemosensitivity of SCLC.	Zheng et al. (2021)
hnRNPA1 R218me2s + R225me2s	*PRMT5*	mTOR inhibitors resistant glioblastomas	In this study, PRMT5 activity (but not expression) was upregulated in response to mTOR inhibitors (rapamycin or PP242). Furthermore, it was revealed that in response to mTOR inhibition, the activated PRMT5 dimethylated hnRNPA1, which resulted in stimulation of hnRNPA1 binding to cyclin D1 and c-MYC internal ribosome entry site (IRES) RNAs. This promoted mRNA translation of the latter two, resulting in drug resistance in glioblastomas	Holmes et al. (2019)
BAG family molecular chaperone regulator 5 (BAG5) R15me2a + R24me2a	*PRMT6*	Sorafenib resistant hepatocellular carcinoma	In this study PRMT6 physically interacted with BAG5, and methylated the N-terminal region of this protein at R15 and R24. Methylation of BAG5 enhanced interaction of this protein with its interacting partner Heat shock cognate 71 kDa protein (HSC70), a well- known autophagy player, leading to degradation of HSC70. Deficiency of PRMT6 induced autophagy to promote tumorigenicity, cell survival and sorafenib resistance in hostile microenvironments of HCC tumors by regulating BAG5-associated HSC70 stability through post-translational methylation of BAG5. BAG5 inhibition increased sensitivity of the HCC cells to sorafenib treatment	Che et al. (2021)
p21 R156me2a		Doxorubicin resistant CRC	Methylation of p21 promotes phosphorylation of p21 at threonine 145, resulting in the p21 to move from the nucleus into the cytoplasm. Translocation of p21 into the cytoplasm by PRMT6 resulted in reduced DOX sensitivity due to changes in cell cycle progression and apoptosis	Nakakido et al. (2015)

One or more CH_3_ groups are predominantly added to lysine (K) and arginine (R) residues giving rise to N-methylation. However, several other amino acids can undergo N-, S- or O- methylation, with methionine (M) and cysteine (C) undergoing S-methylation, aspartate (D) and glutamate (E) undergoing O-methylation and lastly, asparagine (N), histidine (H), proline (P) and alanine (A) undergoing N-methylation (Deng et al., 2016; Cloutier et al., 2013; Clarke, 2013). Methylation does not alter the overall charged state of the residue; however, it increases the hydrophobicity and bulkiness of the protein. This can affect protein-protein interactions and recognitions, which in turn alters gene regulation (Kaniskan H. U. et al., 2015).

### 2.1 Protein methyltransferases (PMTs)

PMTs are responsible for catalysing the transfer of a CH_3_ group to methylated or unmethylated lysine or arginine residues from the methyl donor SAM (Levy, 2019). Each PMT within its respective family is structurally distinct, with the main differences being dependent on the amino acid sequences they recognise for catalysis, the residues (or side chains) they methylate and their 3D structure (Richon et al., 2011). However, a common feature is the overall structure of their catalytic active site composed of a SAM-binding pocket and a residue acceptor channel (Copeland et al., 2009). These two sites are linked together by a hydrophobic channel, which permits the transfer of a CH_3_ group from the SAM onto the amino acid through a state known as the S_N_2 transition. This gives rise to the methylated product and the release of S-5′-adenosyl-L-homocysteine (SAH), the cofactor product (Kaniskan et al., 2018). The vast majority of the currently identified PMTs are categorised into three superfamilies, according to their overall structure and sequence similarities, namely, the Seven-β-strand (7βS) domain family, the SpoU-TrmD (SPOUT) domain family and the Su(var)3-9, enhancer of zeste (E(z)), and trithorax (trx) (SET) domain family (Clarke, 2013).

The 7βS family is the most diverse family among proteomes, with around 125 enzymes harbouring this domain that features a Rossmann-like structural core and can methylate amino acids, RNA and DNA (Petrossian and Clarke, 2010; Carlson and Gozani, 2016). The SPOUT methyltransferase family is the second largest SAM-dependent enzyme family within all proteomes containing a distinctive α/β knot-like structure and methylating RNA substrates (Petrossian and Clarke, 2010; Tkaczuk et al., 2007; Clarke, 2013). The SET domain family is the largest family in humans with around 56 SET domain-containing methyltransferases being identified to date (Clarke, 2013). It contains folds of multiple tiny β-sheets, which encircle into a knot-like conformation, wrapped with a pre- and post-SET domain. An i-SET domain is found within the SET domain itself and all domains are essential for enzymatic activity (Cheng et al., 2005; Kaniskan et al., 2018). Additionally, the SET proteins also contain four conserved sequence motifs, SET motif I (GxG), SET motif II (YxG), SET motif III (RFINHxCxPN), and SET motif IV (ELxFDY), where each motif is involved in the methylation reactions (Cheng et al., 2005).

Some PMTs can perform one round of catalysis, while some can perform multiple rounds. A processive mechanism is when multiple CH3 groups are added to the protein before the protein dissociates from the enzyme. On the other hand, if the protein is dissociated after the first CH_3_ group is added, this is referred to as a distributive mechanism. With the latter mechanism, the methylated protein can rebind to the same or different PMT for additional methyl modifications (Copeland et al., 2009; Al-Hamashi et al., 2020; Fulton et al., 2018). Both PKMTs and PRMTs make use of these two mechanisms.

For the scope of this review the focus will be on the two major groups of PMTs, based on which residue they modify, i.e., protein lysine methyltransferases (PKMTs), which are responsible for methylating lysine residues, and protein arginine methyltransferases (PRMTs), which methylate arginine residues (Tables 1, 2) (Al-Hamashi et al., 2020; Fulton et al., 2018).

#### 2.1.1 Protein lysine methyltransferases (PKMTs)

Lysine (K) residues can be mono-, di- or tri-methylated on their epsilon (Ԑ)-amine group (Greer and Yang, 2012). Lysine methylation partakes in regulating gene expression via histone methylation, which can activate or repress transcription depending on which lysine residues are methylated and the degree of methylation. For example, mono-methylation of Histone 3 Lysine 4 (H3K4me1), mono-methylation of Histone 3 Lysine 27 (H3K27me1), tri-methylation of Histone 3 Lysine 36 (H3K36me3) and tri-methylation of histone 3 lysine 79 (H3K79me3) are linked to transcription activation, while di- or tri-methylation of Histone 3 Lysine 9 (H3K9me2 and H3K9me3) and tri-methylation of Histone 3 Lysine 27 (H3K27me3) are linked to transcription repression (Barski et al., 2007). Overall, lysine methylation is responsible for PTM crosstalk, subcellular localisation, protein stability, DNA promoter binding affinity alteration and protein-protein interactions (Cornett et al., 2019; Hamamoto et al., 2015).

The PKMTs are generally subdivided into two groups, the SET-domain containing and the non-SET domain containing enzymes (Kaniskan et al., 2018). The disruptor of telomeric silencing-1-like (DOT1L) enzyme is the only member of the latter group (Kaniskan et al., 2018; Richon et al., 2011). DOT1L is similar in structure to the PRMT enzymes (Type I) (as will be explained in Section 2.1.2. It is composed of an N-terminal helical domain and a 7βS harbouring the methyl donor binding site and an active site pocket covered with conserved hydrophobic residues (Falnes et al., 2016). The core of the DOT1L also contains seven conserved sequence motifs (I, II, III, IV, VI, VIII and X), which are scattered across the conserved core (Cheng et al., 2005). There are other PKMTs bearing the 7βS domain, known as the methyltransferase-like (METTL) proteins, however this review will not tackle this group (Falnes et al., 2016; Wong and Eirin-Lopez, 2021).

The SET domain PKMTs are further distributed among eight groups (KMT1-KMT8) based on the sequence similarities surrounding the SET domain, their enzymatic activity and their methylation targets (Allis et al., 2007; Kaniskan et al., 2018). Most of the SET domain enzymes primarily methylate histones, some can methylate both histones and non-histone proteins, while very few primarily methylate non-histones. However, there is still quite a large number of SET-domain enzymes that have no known substrates and their enzyme activity is still uncertain (Carlson and Gozani, 2016). As a result of this, it is difficult to precisely sort SET domain enzymes based on their substrates and activity on protein substrates, despite these two factors being considered when the eight KMT sub-groups were created.

#### 2.1.2 Protein arginine methyltransferases (PRMTs)

Arginine (R) residues can be monomethylated (MMA) or di-methylated either symmetrically (me2s or SDMA) or asymmetrically (me2a or ADMA) on the guanidyl group (Greer and Yang, 2012). Arginine methylation has been observed mostly on H3 (H3R2me2a, H3R2me2s, H3R8me2s, H3R8me2a, H3R17me2a and H3R26me2a) and H4 (H4R3me2a, H4R3me2s and H4R17me), which in turn regulates transcription. Histone arginine methylation can activate or repress the expression of tumour suppressor proteins, depending on which histones are methylated. For example, methylation of H3R8 (H3R8me2s) and H4R3 (H4R3me2s) by PRMT5 results in silencing of the tumour suppressor *Rb1* (Wang et al., 2008). In general, transcription activation in linked to ADMA residues while SDMA residues are associated with transcriptional repression. Having said that, there are exceptions where this is not always the resulting outcome (Bedford and Clarke, 2009; Yang and Bedford, 2013). Moreover, arginine methylation is involved in RNA processing and transcriptional elongation, cell cycle and DNA damage repair, apoptosis and signal transduction (Blanc and Richard, 2017; Clarke, 2013; Wei et al., 2014).

A total of nine human PRMTs have been identified and studied to date, with these being divided into three sub-types: Type I, Type II and Type III. All three sub-types can give rise to MMA, however Type I and Type II PRMTs can also give rise to ADMAs and SDMAs, respectively. PRMTs are found in the nucleus or cytoplasm, except for PRMT8, which is predominantly found on the plasma membrane. Among the known PRMTs, PRMT1 was the first one to be identified and thus is the most extensively studied, mediating the majority of arginine methylation events taking place inside cells (Obianyo et al., 2011).

The Type I PRMTs include PRMT 1, 2, 3, 6, 8 and Coactivator-Associated Arginine Methyltransferase 1 (CARM1; PRMT4), the Type II enzymes include PRMT5 and 9, while PRMT7 falls under Type III (Al-Hamashi et al., 2020; Fulton et al., 2018). Normally, Type II PRMTs (PRMT5) and some Type I PRMTs (except PRMT8) can methylate both histones and non-histones, with the majority methylating glycine and arginine rich (GAR/RGG/RG) motifs (Al-Hamashi et al., 2020). However, CARM1 exclusively methylates proline-, glycine-, and methionine-rich (PGM) motifs. Some PRMTs can also read their own unique substrate motifs. For instance, PRMT5 can recognise PGM motifs, while PRMT7 can read RXR sequences (X is equivalent to any amino acid) (Kaniskan et al., 2018; Al-Hamashi et al., 2020).

The PRMT family shares one methyltransferase (MTase) domain (except PRMT7 which has two), a 7-β-barrel Rossmann fold domain (interrelates with SAM) and a dimerization arm (Kaniskan et al., 2018; Wei et al., 2014; Al-Hamashi et al., 2020). The MTase domain is highly conserved and is composed of a THW loop and three motifs; motif I (VLD/EVGXGXG), post I (V/IXG/AXD/E), motif II (F/I/VDI/L/K) and motif III (LR/KXXG) (Wei et al., 2014). Further, some PRMTs have additional domains, such as PRMT2 having a SRC homology 3 (SH3) domain, PRMT3 and 9 having a zinc finger domain, PRMT9 having an F-box domain, PRMT9 having a tetratricopeptide repeat 2 (TPR2) domain and PRMT8 having an N-terminal myristoylation (myr) motif (Wei et al., 2014).

### 2.2 PMTs in cancer

PMTs are implicated in important cellular processes such as gene expression, cell proliferation, growth and differentiation. Dysregulation in PMT expression and activity interferes with such signalling pathways and cellular functions, giving rise to certain diseases like cancer. PMT activity in carcinogenesis and metastasis is affected by point mutations, gene rearrangements and gene amplifications (Richon et al., 2011; Au et al., 2013; Morin et al., 2010). Moreover, the involvement of PMTs in cancer development has become a hot topic in the methyl proteome field in recent years, with various reviews already published in this aspect (Bennett et al., 2017; Huang et al.,2017; Hamamoto and Nakamura, 2016; Husmann and Gozani, 2019; Hwang et al., 2021; Wu et al., 2021). More recently, research has also shifted towards investigating how PMTs are contributing to cancer chemoresistance development, an area which is still underexplored. Certain methylation histone and non-histone markers arising due to PMTs dysregulation have already been reported to be contributing to cancer chemoresistance development (Yang et al., 2020; Zhang L. et al., 2024; Zhu et al., 2023), as summarised in Tables 1, 2 below. However, how PMTs are contributing to chemoresistance development and how this phenomenon can be reversed will be further discussed in Section 3.1.

## 3 Chemoresistance

The development of chemoresistant tumours remains one of the main challenges of cancer treatment. Resistance to therapeutic agents in cancer patients can be classified as inherent or acquired (Mansoori et al., 2017). Inherent resistance refers to genetic characteristics within the cancer cell that provide a protective mechanism for survival, making the therapy ineffective (Mansoori et al., 2017). In contrast, acquired resistance develops after drug exposure in tumours that were initially sensitive (Mansoori et al., 2017). Further, tumours contain a high degree of molecular heterogeneity, thus even a small number of resistant cancer cells or the presence of cancer stem cells (CSC) can give rise to a chemoresistant phenotype (Mansoori et al., 2017; Phi et al., 2018).

There are several mechanisms of drug resistance. For instance, chemotherapeutic agents can induce tumour cells to express more of the therapeutic target or activate other compensatory signalling pathways such as the phosphoinositide 3-kinase (PI3K) pathway or mitogen-activated protein kinase (MAPK) signalling (Yeldag et al., 2018). Additionally, cancer cells can actively remove the drug by expressing ATP binding cassette (ABC) transporters on the cell membrane (Sun et al., 2012). The Epithelial-to-Mesenchymal transition (EMT) programme enables epithelial cells to lose cell polarity and cell-to-cell adhesion to gain a mesenchymal, stem cell-like phenotype. These mesenchymal cells show higher resistance to chemotherapy, in various cancers (Creighton et al., 2009; El Amrani et al., 2019).

When it comes to the role of PMTs in relation to altered gene expression in cancer cells, alternative splicing is one of the mechanisms tumours employ to gain drug resistance and this is achieved either through mutations in intragenic regions or altered expression of splicing factors and splicing patterns (Bonner and Lee, 2023). Dysregulated splicing factors in solid tumours include multiple serine and arginine-rich (SR) family proteins (particularly Serine/arginine-rich splicing factor 1; SRSF1), heterogeneous nuclear ribonucleoproteins (hnRNPs), and members of other RNA binding proteins (including RBM5, RBM10, and RBFOX2) (Bradley and Anczuków, 2023). Arginine methylation, particularly by Type 1 PRMTs and PRMT5 on such proteins has been shown to play a key role and its inhibition has shown promise in pre-clinical therapeutic testing (Fong et al., 2019).

### 3.1 PMTs in cancer chemoresistance

Upon development of acquired chemoresistance, the therapeutic drugs lose their effectiveness or do not kill all the cancer cells, leading to proliferation of the remaining cells. Dysregulation of protein methylation and the PMTs that bring it about, is one of the mechanisms involved in drug resistance. This section together with Tables 3, 4 focus on recent studies concerning the main PKMTs and PRMTs that are involved in chemoresistance or the mechanisms underlying chemoresistance. However, whether any methylation substrate is targeted by said PMTs in these studies is unknown. Moreover, Table 5 summarises and focuses on the completed/ongoing studies on the use of PMT inhibitors in reversing cancer chemoresistance.

**TABLE 3:** The role of PKMTs in cancer drug resistance.

PKMT	Treatment/Cancer	Mechanism of resistance	References
EZH2	Doxorubicin and paclitaxel resistant breast cancer	Activation of STAT3 signalling Upregulation of Notch and Wnt/β-catenin pathway	Yang et al. (2021)
	Cisplatin resistant gastric cancer	Downregulation of PTEN	Li et al. (2019a)
G9a or EHMT2	Imatinib resistant leukemia stem cells	Repression of *SOX6* expression	Zhou M et al. (2021)
	Erlotinib resistant NSCLC	Upregulation of p-Akt Downregulation of PTEN expression	Wang et al. (2018a)
SETDB1	Gemcitabine resistant bladder cancer	Activation of Wnt/β-catenin pathway Increased RAD51 and RRM1 expression	Meng et al. (2023)
	5-FU resistant CRC	Inhibition of TP53 expression	Chen et al. (2017)
SYMD3	Cisplatin resistant NSCLC	Upregulation of CDKs	Lv et al. (2021)
	Cisplatin resistant breast cancer	Downregulation of miR-124	Wang et al. (2020b)
SYMD2	Multidrug resistance (5-FU, doxorubicin, cisplatin and sunitinib) in RCC	Upregulation of P-gp	Yan et al. (2019)
	Oxaliplatin resistance in colon adenocarcinoma	Upregulation of P-gp Activation of MEK/ERK/AP-1 signalling pathway	Ren et al. (2019)
	Temozolomide and cisplatin resistance in glioma	Upregulation of EMT markers (N-cadherin, COL1A1) Inhibition of P53 pathway	Pan et al. (2022)
SETD7/9	Doxorubicin and etoposide resistance in NSCLC	Regulation of cyclins	Daks et al. (2021)
SETD1A	Sorafenib resistance in hepatocellular carcinoma	Activation of YAP signalling	Wu et al. (2021)
	Tamoxifen resistance in breast cancer	Increase in SOX2 expression	Jin et al. (2022)
SUV39H2	Docetaxel resistance in prostate cancer	Increase in tumour stemness and CSC markers (*SOX2, OG, CD44, CD133*, and *OCT4*) Activation of Akt/FOXO pathway	Sun et al. (2024)

**TABLE 4:** The role of PRMTs in cancer drug resistance.

PRMT	Treatment/Cancer	Mechanism of resistance	References
PRMT1	Olaparib resistance in breast cancer cells	Upregulation of c-MYC Enhances DNA repair	Hsu et al. (2021)
	Cisplatin resistance in tumour initiating cells of ESCC	Activation of Wnt/Notch pathway	Zhao M et al. (2021)
	Gemcitabine resistant PDACs	Epigenetic regulation of the MAFF/BACH1 regulatory axis	Nguyen et al. (2024)
PRMT5	Doxorubicin resistance in breast cancer cells	Increased CSCs Upregulation of OCT4/A, KLF and c-MYC	Wang et al. (2018b)
	Cisplatin resistance in NSCLC cells	Upregulation of P-gp and MRP-1 Inhibition of miR-138-5p expression Upregulation of MYH9	Xu et al. (2021)
	Gemcitabine resistant PDACs	Depletion of UBR7 Increased the stability of PRMT5 Increase stimulation of glycolysis	Bi et al. (2023)

**TABLE 5:** Current inhibitors developed against PMTs which have shown potential in reversing chemoresistance at a pre-clinical or clinical level.

PMTs inhibitors investigated at a pre-clinical level using cell lines or xenografts					
PMT Target/s	Inhibitor name	Treatment + cancer	Experimental model	Role in drug resistance	References
*G9a/EHMT2*	UNCO638	Erlotinib resistant NSCLC	1) PC9 + HCC827 cell lines 2) PC9/Erlotinib SCID mice	G9a inhibition promoted the re-expression of the PTEN gene, attenuating AKT signalling and reversing erlotinib resistance	Wang et al. (2018a)
		Gemcitabine resistant Pancreatic Cancer Cells	1 PANC-1 and Mia-paca-2 cell lines 2 PANC-1 gemcitabine-resistant cells inoculated in the pancreas of xenograft mice	G9a inhibition reduced the production of IL-8, thereby reducing the activation of pancreatic stellate cells which are responsible for gemcitabine resistance	Pan et al., 2016
	UNC0642	Olaparib (PARi) resistant Ovarian Cancer	1) PEO1 cell line 2) NOD SCID gamma (NSG) mice with PARPi-resistant high-grade serous ovarian carcinoma cells (HGSOC)	Disrupting EHMT2 re-sensitizes HGSOC to PARPi (Olaparib), through DNA damage and cell cycle dysregulation	Watson et al. (2019)
*EZH2*	Tazemetostat (EPZ6438)	DOX or Paclitazel resistant Breast cancer	1) CAL51 MDA-MB-231 or MCF7 cell lines 2) CAL51/DOX xenografts	Tazemetostat reversed the acquired chemoresistance arising through the EZH2/STAT3 axis, which secreted chemotherapy-elicited exosomes enriched in miR-378a-3p and miR-378d	Yang et al. (2021)
	EPZ011989	Cisplatin, etoposide or irinotecan resistant SCLC	1) 11 different chemosensitive or chemoresistant PDXs	Preventing silencing of *SLFN11* (Table 1) by this inhibitor prohibited emergence of acquired resistance and augmented chemotherapeutic	Gardner et al. (2017)
	GSK126 (GSK2816126)	Oxaliplatin resistant CRC	1) HCT116 Cell Line 2) HCT116/Oxaliplatin BALB/c (nu/nu) nude male mice	Increased the expression of MEISI (Table 1) and resensitised resistant CRC cells to oxaliplatin	Li et al. (2022)
*SMYD2*	BAY-598	Cisplatin resistant NSCLC	1) A549 and NCI-H460 Cell Lines 2) NCI-H460/Cisplatin xenograft male BALB/C mice	SMYD2 inhibition enhanced p53 pathway activity and induced cell apoptosis	Shang and Wei (2019)
	AZ505				
*SETDB1*	Mithramycin	Cetuximab resistant CRC	1) LoVo and SW480 cell lines 2) SW480 cells in Female BALB/c nude mice	SETDB1 inhibition attenuated PI3K/AKT signalling in the KRAS-mutated CRC cells	Hou et al. (2020)
*PRMT1*	MS023	Cisplatin resistant ovarian cancer	1) SK-OV-3 cell line	Abrogated cisplatin induced SASP gene expression	Musiani et al. (2020)
	DB75	Gemcitabine resistant PDAC	1) MDA28, MDA48, PANC-1, L3.5, L3.7, MiaPaCa-2, BxPC-3 cell lines 2) H7 mouse PDAC cells in C57BL/6 mice	Interrupts the methylation of HSP70 by PRMT1 (Table 2), thus preventing binding between methylation HSP70 and BCL-2 mRNA through AREs in its 3′-UTR.	Wang et al. (2020a)
	TC-E5003		1) L3.5, L3.7, MiaPaCa-2, BxPC-3 cell lines		
*PRMT3*	SGC707	Gemcitabine resistant PDAC	1) PANC-1 cell Line	SGC707 significantly reduced the expression of ABCG2, which resensitised the cells to gemcitabine	Hsu et al. (2018)
*PRMT5*	EPZ015666	mTOR (PP242) resistant glioblastomas	1) LN229, LN18, GBM6 and GBM39 cell lines 2) LN229 female C.B.-17-scid (Taconic) mice	Prevents PRMT5 dimethylated hnRNPA1, thus inhibiting stimulation of hnsRNPA1 binding to cyclin D1 and c-MYC internal ribosome entry site (IRES) RNAs	Holmes et al. (2019)
*PRMT5*	DS-437	Anti-erbB2 targeted antibody therapy and regulatory T-cells	1) Drug-resistant syngeneic murine breast cancer (CT26-Her2 cells) model	Decreased the activity of tumour-specific T_reg_ cells in a FOXP3-dependent manner	Nagai et al. (2019)
		Gemcitabine resistant PDAC	1) PDX models of PDAC with or without gemcitabine resistance	PRMT5 inhibition reduced gemcitabine resistance in pancreatic cancer caused by UBR7 depletion	Bi et al. (2023)
	PRT543	Ibrutinib resistant Lymohomas	1) MCL Cell Lines 2) Mouse model of ibrutinib-resistant mantle cell lymphoma (preclinical MCL models)	Disruption of constitutive PI3K/AKT signaling, dephosphorylation and nuclear translocation of FOXO1, and enhanced recruitment of this tumor suppressor protein to chromatin	Brown et al. (2019)
*G9a/EHMT2 and EZH2*	SU08	Cisplatin or paclitaxel or erlotinib resistant NSCLC	1) H1299, H460, A549, PC9 cell lines 2) H460/Cisplatin or PC9/Erlotinib male BALB/c nude mice	Dual inhibition of EZH2 and G9a by SU08, demonstrated re-sensitization to three different therapies by regulating the SMAD4/ERK/c-Myc signalling axis	Zhang et al. (2024b)
*Type I PRMT inhibitors*	MS023	Talazoparib (BMN-673 - PARPi) resistant ovarican cancer	1) PEO1 and PEO4 cell lines	Hypothesised that MS023 creates/restores homologous recombination defects, thus making the cancer cells vulnerable to PARPi	Dominici et al. (2021)
	GSK3368715	Gemcitabine resistant pancreatic ductal adenocarcinomas	1) Panc1 (female), AsPC1 (female), MiaPaCa2 (male), HPAFII (male), CAPAN-1 (male) and mT4 (murine) PDAC cells 2) Human PDAC PDXs	Prevents PRMT1 from inhibiting chemo-induced chromatin recruitment of the MAFF/BACH1 complex	Nguyen et al. (2024)

PMTs Inhibitors investigated at a Clinical Level Using Patients					
PMT Target/s	Inhibitor Name	Treatment + Cancer	Clinical Stage	Status	Trial ID
*EZH2*	Tazemetostat (EPZ6438)	Pembrolizumab (PD-1 blockade) NSCLC	Phase Ib and II	Ongoing (2028 estimated completion)	NCT05467748
		Resisted treatment (refractory) lymphoma	Phase I	Ongoing (2025 estimated completion)	NCT05627245
	PF-06821497 (Mevrometostat by Pfizer)	Castration resistant prostate cancer resistant to SOC and Refractory Small Cell Lung Cancer (SCLC)	Phase I	Ongoing (2025 estimated completion)	NCT03460977
*PRMT5*	AZD3470	Relapsed/Refractory Haematologic Malignancies	Phase I/II	Ongoing (2026 estimated completion)	NCT06137144

#### 3.1.1 PKMTs and drug resistance

##### 3.1.1.1 EZH2

The Enhancer of zest homolog 2 (EZH2) is responsible for catalysing the mono-, di- and tri-methylation of H3K27 that are associated with transcriptional repression. EZH2 has been known to be significantly overexpressed in drug-resistant cancer cells of multiple myeloma, leukemia, breast, colorectal (CRC), prostate and ovarian tumours (Li et al., 2017; Li Q. et al., 2019; Li X. et al., 2021; Rastgoo et al., 2018; Wen et al., 2021; Yang et al., 2021). For instance, Zhou M et al., 2021 showed that oxaliplatin resistance in CRC arises from high EZH2 protein expression. Here, the tripartite motif containing 25 (TRIM 25) prevented the ubiquitination and degradation of EZH2 (Zhou S et al., 2021).

Although the exact mechanism behind the involvement of EZH2 in drug resistance is unclear, many studies reported the role of EZH2 in maintaining CSCs through the modulation of proteins such as Signal transducer and activator of transcription 3 (STAT3) and key pathways like the Wingless-related integration site (Wnt)/β-catenin pathway. Indeed Yang et al. (2021) revealed that the increased expression of EZH2 in both doxorubicin- and paclitaxel-resistant breast cancer cells activated STAT3 signalling, which promoted the expression of miR-378a-3p and miR-378d and induced their exosomal secretion. The miRNAs targeted the WNT antagonist Dickkopf WNT signaling pathway inhibitor 3 (DKK3) and the Notch suppressor Endocytic Adaptor Protein (NUMB) in the cancer cells that remained viable following chemotherapy. In this way, the miRNAs modulated the WNT/β-catenin and Notch signalling pathways, both of which are linked with CSC maintenance and drug resistance.

In osteosarcoma, circular PR domain zinc finger protein 2 (circPRDM2) positively modulated EZH2 expression in doxorubicin-resistant cells by targeting miR-760, inhibiting its tumour suppressive roles (Yuan et al., 2021). However, the relationship between circPRDM2, EZH2 and miR-760 in chemoresistance is unclear and requires further investigation. Furthermore, the long non-coding RNA (lncRNA) prostate cancer-associated transcript 1 (PCAT-1) was found to be upregulated in cisplatin resistant gastric cancer cells (Li H. et al., 2019). Li et al. (2019a) found that PCAT-1 recruited EZH2, which epigenetically supressed the expression of the tumour suppressor Phosphatase and tensin homolog (PTEN) by increasing H3K27me3 levels.

In another study, EZH2 interacted with DNMT3a and was proven to contribute to oxaliplatin resistance in CRC by epigenetically silencing and inhibiting the expression of MEIS1 (Table 1) (Li et al., 2022). Additionally, EZH2 was also found to bind to the Euchromatic histone-lysine N-methyltransferase 2 (EHMT2) driving cisplatin and paclitaxel resistance in NSCLC cells (Zhang Q. et al., 2024). EZH2 was found to work with EHMT2 to silence the expression of the tumour suppressor of SMAD family member (SMAD) 4 which in turn activates the extracellular signal-regulated kinase (ERK)/cellular myelocytomatosis oncogene (c-MYC) pathway, controlling drug resistance (Table 1) (Zhang Q. et al., 2024).

##### 3.1.1.2 Euchromatic histone-lysine N-methyltransferase 2 (EHMT2)/G9a

Euchromatic histone-lysine N-methyltransferase 2 (EHMT2), also known as G9a, modifies H3K9 together with SETDB1, Suv39H1 and Suv39H2. Specifically, G9a catalyses the formation of H3K9me1 and H3K9me2 which results in gene expression repression, apart from also methylating other lysine residues on H3).

Several studies report the role of G9a in cancer drug resistance. For instance, G9a was shown to be significantly upregulated in patients with resistant acute myeloid leukemia (AML) in comparison with the complete remission group (Gouda et al., 2022). In chronic myeloid leukemia (CML) stem cells (LSCs) resistant to imatinib, G9a was also found to be overexpressed and responsible for repressing the transcription of the tumour suppressor SRY-Box Transcription Factor 6 (SOX6) (Zhou M. et al., 2021).

Moreover, G9a was found to be overexpressed in erlotinib resistant (i.e., Epidermal growth factor receptor (EGFR)-tyrosine kinase inhibitor (TKI) resistant) non-small cell lung cancer (NSCLC) cells (Table 1) (Wang L. et al., 2018). In these cells, G9a epigenetically inhibited PTEN transcription by depositing H3K9me2 at its promoter region (Wang L. et al., 2018). This resulted in the activation of PI3K/AKT signalling which promoted the migration and self-renewal capacity of the NSCLC cells (Wang L. et al., 2018). In contrast, in another study on NSCLC, G9a was found to have an opposing effect. In this case, G9a prevented paclitaxel resistance by transcriptionally repressing the levels of aldehyde dehydrogenase 2 (ALDH2) and G9a inhibition upregulated ALDH2 levels (Wang W. et al., 2022). ALDH2 is a CSC marker associated with tumour cell proliferation and paclitaxel resistance in NSCLC cells (Wang W. et al., 2022). Hence, the role of G9a in NSCLC chemoresistance depended on which gene it transcriptionally repressed.

##### 3.1.1.3 SETDB1

SETDB1 is responsible for the di–and tri-methylation of H3K9, which is associated with gene silencing. It is involved in the methylation of many key proteins and signalling cascades implicated in oncogenesis and drug resistance such as p53, alpha serine/threonine-protein kinase or protein kinase B (AKT), Wnt/β-catenin, Janus tyrosine kinase (JAK)/STAT3 and SMAD2-3 (Chen et al., 2017; Hou et al., 2020; Liu et al., 2022; Meng et al., 2023).

Increased expression of SETDB1 is found in several drug-resistant cancer cells. For instance, the circular non-coding RNA generated from the SETDB1 mRNA, serum circSETDB1, was found to be upregulated in ovarian cancer patients that displayed lymph node metastasis and resistance to platinum-taxane combined chemotherapy (Wang et al., 2019). Additionally, Meng et al. (2023) demonstrated how the protein tyrosine kinase 2 (PTK2)- Polyadenylate-binding protein 1 (PABPC1) complex stabilised the SETDB1 mRNA, preventing its degradation and hence promoting its protein expression in gemcitabine resistant bladder cancer cells. The increased SETDB1 expression was then responsible for facilitating EMT by activating the Wnt/β-catenin pathway, contributing to cancer progression (Meng et al., 2023). Gene set enrichment analysis revealed that SETDB1 is strongly linked to DNA repair and drug resistance. Further analysis revealed that SETDB1 increases the expression of RAD51 recombinase (RAD51), a protein involved in homologous recombination and ribonucleotide reductase catalytic subunit M1 (RRM1) which contributes to gemcitabine resistance (Meng et al., 2023).

SETDB1 was also found to be overexpressed in cetuximab resistant CRC cells, where it promoted AKT activation (Table 2) (Hou et al., 2020). Inhibition of SETDB1 then limited AKT hyperactivation and reversed cetuximab resistance, even in Kirsten rat sarcoma virus (KRAS)-mutated CRC cells (Hou et al., 2020). In agreement, Chen et al. (2017) showed that SETDB1 expression levels were correlated to poor prognosis in CRC patients and its overexpression suppressed apoptosis induced by 5-FU. SETDB1 inhibited the expression of *TP53* by methylating its promoter, promoting cell survival in the presence of 5-FU (Chen et al., 2017). Similarly, in breast cancer cells, the interaction of SETDB1 and PELP1 was necessary to promote AKT methylation and activation, which in turn led to tamoxifen therapy resistance (Table 2) (Liu et al., 2022).

##### 3.1.1.4 SYMD3

A notable number of research studies identified the role of SYMD3 in drug resistance. For example, SYMD3 was found to control the alkylation damage response in small cell lung cancer (SCLC) (Lukinovic et al., 2022). SYMD3 methylated and activated the E3-ubiquitin ligase RING finger protein 113A (RNF113A), which has been linked to alkylation damage repair, thus inducing resistance against alkylating chemotherapy (Table 2) (Lukinovic et al., 2022). Additionally, it was correlated with cisplatin treatment resistance in NSCLC and breast cancer (Lv et al., 2021; Wang et al., 2020b). In NSCLC, the interaction between SMAD3 and Ankyrin Repeat and KH Domain Containing 1 (ANKHD1) was shown to be essential for the upregulation of cyclin dependant kinases (CDKs) and in particular CDK2, which was found to confer cisplatin resistance (Lv et al., 2021). In breast cancer, SYMD3 negatively regulated miR-124, which is involved in cancer cell sensitivity to cisplatin (Wang et al., 2020b).

##### 3.1.1.5 SYMD2

SYMD2 is involved in cancer cell proliferation, migration, apoptosis and drug resistance (Pan et al., 2022). SYMD2 was found to promote the expression of P-glycoprotein (P-gp) also known as multidrug resistance protein 1 (MDR1) or ATP binding cassette B1 (ABCB1) in renal cell carcinoma (RCC) and CRC cells. (Yan et al., 2019). In addition, in RCC, SYMD2 was found to promote the expression of miR-125b, in turn reducing the levels of DKK3. Inhibition of SYMD2 in RCC cells and in xenograft tumour models through the inhibitor AZ505 sensitised the cancer cells to cisplatin, doxorubicin, 5-FU and sunitinib confirming its role in chemoresistance (Yan et al., 2019). Then in CRC, SYMD2 regulated P-gp expression by activating the Mitogen-activated protein kinase (MEK)/Extracellular signal-regulated kinase (ERK)/activator protein 1 (AP-1) signalling pathway, promoting oxaliplatin resistance (Ren et al., 2019).

Pan et al. (2022) showed that inhibiting SMYD2 by AZ505 (a SYMD2 inhibitor) in glioma cells leads to a reduction in EMT markers such as N-cadherin and Collagen 1A1 (COL1A1). It also activated the expression of genes and proteins involved in the p53 signalling pathway, as evidenced by the upregulation of *p21*, Growth arrest and DNA damage-inducible protein (*GADD45)* and Bcl-2-associated protein x (*Bax)*, along with an increase in H2A histone family member X (*γH2AX*), a marker of DNA damage, and a higher Bax/B-cell leukemia/lymphoma 2 protein (*Bcl-2*) ratio. In contrast, cyclin D1 was downregulated. Additionally, genes related to steroid metabolism, including LDL receptors *(LDLR)* were also downregulated. These molecular changes collectively enhanced the sensitivity of glioma cells to temozolomide and cisplatin (Pan et al., 2022).

##### 3.1.1.6 Other PKMTs

In addition to the PKMTs discussed above, others have also been slightly investigated in the context of chemoresistance. In particular, SETD7/9 was found to be implicated in resistance to doxorubicin and etoposide in NSCLC cells (Daks et al., 2021). The loss of SETD7/9 in the cells, increased the levels of cyclins, which may have increased the sensitivity to genotoxic stress by the chemotherapy (Daks et al., 2021). Moreover, in multiple myeloma, SETD8 was associated with melphalan resistance and its expression was found to be significantly higher in relapsed patients than in newly diagnosed patients (Herviou et al., 2021). Then, inhibition of SETD8 in multiple myeloma cells, overcame melphalan resistance and thus improved the treatment for multiple myeloma patients (Herviou et al., 2021). Wu et al. (2020) uncovered the role of SETD1A in sorafenib resistance in hepatocellular carcinoma by activating Yes-associates protein (YAP) signalling. Similarly, SETD1A was also linked to tamoxifen resistance in breast cancer by promoting the expression of sex-determining region Y-box 2 *SOX2* (Jin et al., 2022).

The suppressor of variegation 3–9 homolog 2 (SUV39H2) was identified as a critical enhancer of CSC populations in prostate cancer (Sun et al., 2024). Specifically, the knockdown of SUV39H2 in prostate cancer cells led to a marked reduction in the expression of key stemness markers, including *SOX2, NANOG, CD44, CD133*, and octamer-binding transcription factor 4 *(OCT4)* (Sun et al., 2024). Additionally, SUV39H2 knockdown significantly impaired cell viability in response to docetaxel treatment compared to control cells, indicating that SUV39H2 contributes to docetaxel resistance in prostate cancer (Sun et al., 2024). Furthermore, the knockdown of SUV39H2 resulted in decreased levels of AKT and forkhead box O3a (FOXO3a), highlighting its role in modulating these markers to confer drug resistance (Sun et al., 2024).

#### 3.1.2 PRMTs and drug resistance

##### 3.1.2.1 PRMT1

PRMT1, the major PRMT found in mammals catalyses the methylation of H4R3 together with other non-histone substrates. In cancer, PRMT1 was found to be associated with lower efficacy of chemotherapy (Matsubara et al., 2021; Shimomura et al., 2021). Cervical and ovarian cancer patients with lower PRMT1 levels were likely to benefit more from cisplatin-based chemotherapy and had a longer overall survival period when compared to the patients with high PRMT1 expression (Matsubara et al., 2021; Shimomura et al., 2021). Indeed, the downregulation of PRMT1 in both cervical cancer and ovarian cancer cells improved their sensitivity to cisplatin treatment (Matsubara et al., 2021; Shimomura et al., 2021). Likewise, PRMT1 was found to be overexpressed in triple negative breast cancer (TNBC) and oesophageal squamous cell carcinoma (ESCC) (Suresh et al., 2022; Zhao et al., 2019).

In TNBC, PRMT1 positively regulated c-MYC protein stability, which controls the expression of homologous recombination gene expression, enhancing DNA repair (Hsu et al., 2021). This resulted in the resistance to the poly (ADP-ribose) polymerase (PARP) inhibitor Olaparib, which works by inhibiting DNA repair mechanisms in the TNBC cells (Hsu et al., 2021). PRMT1 was also responsible for promoting the Wnt and Notch pathways in ESCC, leading to increased tumour initiating cells with enhanced self-renewal capabilities and resistance to cisplatin treatment (Zhao et al., 2019). Additionally, in SCLC, SOX2 methylation by PRMT1, promoted its expression and molecular functions in cancer stemness, self-renewal and chemoresistance (Table 2) (Liang et al., 2022). The increased H4R3me2a mark deposited by PRMT1 upon cisplatin treatment induced the expression of senescence-associated secretory phenotype (SASP) genes such as interleukin (IL)1Α, IL-1Β, IL-6, and tumour necrosis factor-alpha (TNFα) (Table 1) (Musiani et al., 2020). These genes protected ovarian cancer cells from DNA damage and apoptosis, promoting cisplatin resistance (Musiani et al., 2020). Lastly, a recent study demonstrated the involvement of PRMT1 in acquired gemcitabine resistance of pancreatic ductal adenocarcinoma (PDAC) cells (Nguyen et al., 2024). Mechanistically, gemcitabine treatment induced translocation of PRMT1 into the nucleus, where its enzymatic activity limited the assembly of chromatin-bound MAF Basic Leucine Zipper Transcription Factor F/BTB and CNC homology 1 (MAFF/BACH1) transcriptional complexes (Nguyen et al., 2024). In this study, no direct ADMA modifications of MAFF and BACH1 were detected, thus it was hypothesised that PRMT1 indirectly regulates the formation of MAFF/BACH1 complex and its chromatin recruitment. It remains unknown what additional factors together with PRMT1 modulate this complex in response to gemcitabine (Nguyen et al., 2024).

##### 3.1.2.2 PRMT5

PRMT5 is the main type II PRMT, which promotes gene silencing through the methylation of H2AR3, H3R8 and H4R3 (Zhu and Rui, 2019). Like the other PMTs, PRMT5 can also methylate a number of non-histone proteins (Zhu and Rui, 2019).

In glioblastoma, PRMT5 activity conferred therapeutic resistance to mammalian target of rapamycin (mTOR) inhibitors by mediating the methylation of heterogeneous nuclear ribonucleoprotein 1 (hnRNP A1) to promote the translation of cyclin D1 and c-MYC, leading to drug resistance (Table 2) (Holmes et al., 2019). In fact, subsequent inhibition of PRMT5 sensitised the glioblastoma cells to mTOR inhibition (Holmes et al., 2019). In agreement, Wang Z. et al. (2018) found that PRMT5 mediated doxorubicin resistance in breast cancer by inducing the expression of the stem cell markers octamer-binding transcription factor 4 (OCT4/A), Krupple-like factor 4 (KLF4) and c-MYC (Wang Z. et al., 2018). Further, circ-PRMT5 was found to be increased in cisplatin-resistant NSCLC cells (Xu et al., 2021). Its knockdown reduced the expression of the drug transporter P-gp and multidrug resistance-associated protein 1 (MRP-1)and induced an immune response by increasing the expression of IL-2, TNF-a and decreasing the levels of transforming growth factor beta (TGF-β) (Xu et al., 2021). Further analysis revealed that circ-PRMT5 controls these effects by sponging miR-158-5p, supporting the expression of myosin heavy chain 9 (MYH9) which promotes cancer progression (Xu et al., 2021). Lastly, gemcitabine resistance in PDAC patient derived xenografts (PDXs) was demonstrated to mechanistically arise due to depleted UBR7 which resulted in increased stability of PRMT5 and increased glycolysis promotion (Bi et al., 2023).

##### 3.1.2.3 Other PRMTs

Overexpression of other PRMTs has also been implicated in chemoresistance. For instance, the overexpression of PRMT3 led to gemcitabine resistance in pancreatic cancer cells by upregulating the expression of the drug transporter ABCG2 (Hsu et al., 2018) (Table 2). Then, in SCLC, CARM1 was found to regulate SMAD7 methylation to activate TGF-β/SMAD signalling, promoting EMT and chemoresistance (Zheng et al., 2021) (Table 2). In contrast, knockout of CARM1 induced drug resistance in breast cancer cell lines whereas high CARM1 expression in breast cancer patients increased their overall survival after adjuvant chemotherapy (Wang et al., 2015). Finally, PRMT6 has been shown to methylate p21, increasing its cytoplasmic localisation and inhibiting its growth suppressive roles (Nakakido et al., 2015) (Table 2).

## 4 Potential targeting of PMTs to reverse chemoresistance

PMT inhibitors have different modes of action, as well reviewed by Wang and his colleagues (2022). They either compete with SAM, which is the methylation cofactor, occupying the SAM-binding pocket or compete with the substrate and occupy the substrate-binding site of the methyltransferase (Ferreira de Freitas et al., 2019; Wang M. Y. et al., 2022). Cofactor inhibitors are more challenging for drug discovery than substrate competitors due to the hydrophilicity of the SAM-binding pocket (Schapira*,* 2016). Developing compounds that are polar enough for the SAM-binding site but also hydrophobic enough to cross the cell membrane is very difficult (Schapira, 2016). Moreover, the inhibitors have to compete with the high cellular levels of SAM. However, cofactor competitors were the first to reach the market. For instance, the PRMT5 inhibitors LLY-283 and JNJ-64619178 have been the starting point for SAM mimetics with good potency and cellular activity (Wang M. Y. et al., 2022). Others like the SYMD2 inhibitor PFI-5 retain the adenine ring of the cofactor or are chemically unrelated to SAM but still sufficiently inhibit its function (Ferreira de Freitas et al., 2019).

In contrast, the substrate-binding site is less polar than the SAM-binding pocket and is more structurally-druggable, hence the majority of PMT inhibitors are substrate competitors (Schapira*.,* 2016). However, SAM or SAH may be required for the binding of substrate inhibitors to their target enzyme (Ferreira de Freitas et al., 2019). The substrate competitor may either interact directly with the cofactor or the cofactor may allosterically stabilise the substrate-binding pocket (Ferreira de Freitas et al., 2019). For instance, some PKMTs like SETD7 require the binding of SAM to generate the substrate-binding groove (Ferreira de Freitas et al., 2019).

Other PMT inhibitors focused on targeting allosteric sites to modulate PMT function. For example, MAK683 is an EZH2 inhibitor that binds to the embryonic ectoderm development (EED) domain. EZH2 is the catalytic subunit of the Polycomb Repressive Complex 2 (PRC2) that is only active in complexes with EED and SUZ12 polycomb repressive complex 2 subunit (SUZ12). Thus, by targeting the EED domain, MAK683 inhibits the catalytic activity of EZH2 (Schapira*.,* 2016).

Targeting key PMTs in cancer can prevent metastatic growth and reverse drug resistance. Most of the currently developed inhibitors against PMTs have been investigated in there use to prevent cancer growth, with a number of reviews covering the different inhibitors synthesised to date, together with their mode of action (Feoli et al., 2022; Hu et al., 2016; Kaniskan H. U. et al., 2015; Kaniskan et al., 2018; Song et al., 2016; Wang M. Y. et al., 2022; Wu et al., 2021). However, whether such inhibitors could also help in reversing cancer chemoresistance is still being investigated. Moreover, combining PMT inhibitors with other anti-cancer therapeutics can prolong cellular effectiveness, reverse drug resistance and improve the response and overall survival rate of cancer patients. This section will focus on the major PKMT and PRMT inhibitors that have shown outstanding pre-clinical potential or are currently being studied in clinical trials for reversing cancer chemoresistance.

### 4.1 Targeting EZH2

Tazemetostat (EPZ6438) is a SAM-competitive EZH2 inhibitor that has been approved by the FDA for the treatment of epithelial sarcoma characterised by loss of integrase interactor 1/SWI/SNF-related matrix-associated actin-dependent regulator of chromatin subfamily B member 1 (INI1/SMARCB1) and follicular lymphoma (FDA, 2020a; FDA, 2020b). The anti-tumourigenic properties have been studied in several malignancies. For example, in breast cancer xenograft models, Tazemetostat reversed doxorubicin resistance by reducing the number of chemotherapy-induced exosomal miR-378a-3p and miR-378d (Yang et al., 2021). In biliary tract cancer cell lines, Tazemetostat has been demonstrated to effectively reduce levels of H3K27me3 and upregulate the gene expression of fructose-bisphosphatase 1 (FBP1) (Bekric et al., 2023). FBP1 is recognised for its role as a tumour suppressor in some cancers such as cholangiocarcinoma (Zhao et al., 2018). Moreover, Tazemetostat is being investigated in more than 50 clinical trials (ClinicalTrials.gov accessed August 2024). These include nerve sheath tumours (NCT04917042 - ClinicalTrials.gov, 2024L), AT-rich interacting domain-containing protein 1A (ARID1A) mutated malignancies (NCT05023655 - ClinicalTrials.gov, 2024e), patients with relapsed or refractory tumours harbouring EZH2, SWI/SNF related matrix associated actin dependent regulator of chromatin subfamily a, member 4 (SMARCA4) or SMARCB1 mutations (NCT03213665 - ClinicalTrials.gov, 2024m), T-cell lymphoma (NCT05983965 - ClinicalTrials.gov, 2024i), NSCLC in combination with pembrolizumab (NCT05467748 - ClinicalTrials.gov, 2024b), metastatic castration-resistant prostate cancer in combination with Talazoparib (NCT04846478 - ClinicalTrials.gov, 2024d) and combination therapy with belinostat (Histome deacetylases inhibitor) in treating patients with lymphomas that have returned (relapsed) or resisted treatment (refractory) (NCT05627245 - ClinicalTrials.gov, 2024n ). Additionally, the success of Tazemetostat in treating some cancers has led to an ongoing study evaluating its long-term safety in patients who have previously benefited from the therapy (NCT02875548 - ClinicalTrials.gov, 2024j). In a completed clinical trial (NCT02860286 - ClinicalTrials.gov, 2021) involving 74 patients with relapsed or refractory BRCA1-Associated Protein 1 (BAP1)-inactivated pleural mesothelioma, Tazemetostat showed limited efficacy: only two patients had partial responses, and none achieved a complete response (Zauderer et al., 2022). This suggests that tumours with specific biomarkers might benefit more from this therapy (Zauderer et al., 2022).

GSK126 (GSK2816126) is another selective, SAM-competitive small molecule inhibitor of EZH2, which inhibits both wildtype and mutant EZH2 (Gulati et al., 2018). In a phase I clinical trial (NCT02082977 - ClinicalTrials.gov, 2020) which took place between 2014 and 2017, a study was performed to investigate the safety, pharmacokinetics, pharmacodynamics and clinical activity of GSK2816126 in 41 subjects having either relapsed/refractory diffuse large B cell lymphoma, transformed follicular lymphoma, other Non-Hodgkin’s lymphomas, solid tumors and multiple myeloma (Gulati et al., 2018). Due to different adverse events (e.g., fatigue, nausea, anemia and vomiting), as well as insufficient evidence of clinical activity, further clinical investigation of this inhibitor were terminated in this study, and since then, no additional clinical studies were performed. However, in recent years, this inhibitor has shown to also resensitize different resistant cancers to therapy (Li et al., 2022; Zhang Q. et al., 2024). For instance, this inhibitor increased the expression of MEISI (Table 1) and made resistant CRC cells sensitive to oxaliplatin again (Li et al., 2022). Similarly, Zhang and his colleagues (2024) also demonstrated that GSK126 increased the therapy sensitivity to cisplatin or paclitaxel resistant NSCLC. Moreover, in the same study performed (Zhang Q. et al., 2024), a novel inhibitor which targets not only EZH2, but also G9a, was developed to reverse to cisplatin, paclitaxel or erlotinib resistance in NSCLC, named SU08. Molecular docking analysis demonstrated that SU08 accommodates in the substrate-binding cavities of G9a and EZH2, thus acting as a substrate competitor. SU08 could inhibit the enzyme activity of EZH2 and G9a in a concentration-dependent manner, with IC_50_ values close to those of single agents GSK126 (EZH2) and UNC0638 (G9a/EHMT2). Despite demonstrating significant potential under both *in vitro* and *in vivo* conditions (Table 5), it was stated that SU08 also requires extensive further work to improve its drug activity and intensive preclinical studies, as well as its use in other resistant cancers (Zhang Q. et al., 2024).

EPZ011989 is another potent, orally-available EZH2 inhibitor that increased the sensitivity of cisplatin, etoposide and irinotecan in both chemosensitive and chemoresistant models of SCLC (Gardner et al., 2017). Ramakrishnan et al. (2019) also reported that EPZ011989 increased the number of natural killer cells (NKCs) in cisplatin-treated bladder cancer cells. These NKCs have the potential to enhance cisplatin sensitivity by increasing the cytotoxicity of stem-like cells or promoting the differentiation of tumour cells.

PF-06821497, CPI-205, MAK683 and DS-3201 are other EZH2 inhibitors that are currently being studied in clinical trials on different cancer sub-types as a monotherapy or in combination with other therapeutics. For example, PF-06821497 (Mevrometostat by Pfizer) is currently being investigated in patients with advanced prostate cancer in combination with enzalutamide (NCT06551324 - ClinicalTrials.gov, 2024k). Ongoing clinical trials for Mevrometostat are also being carried out for relapsed and refractory SCLC, castration resistant prostate cancer (including those resistant to standard or care (SOC)) and follicular lymphoma (NCT03460977 - ClinicalTrials.gov, 2024c). Recently, a phase I study has been completed for the evaluation of CPI-1205 in 32 patients with B-cell lymphoma (NCT02395601 - ClinicalTrials.gov, 2022d). CPI-1205 was well-tolerated with manageable adverse effects and showed evidence of antitumor activity (Harb et al., 2018). CPI-1205 was also studied in combination with ipilimumab in patients with advanced solid tumours (NCT03525795 - ClinicalTrials.gov, 2022b) and advanced prostate cancer in combination with enzalutamide or abiraterone/prednisone (NCT03480646), however, despite both studies having concluded, the results have not yet been published or made publicly available. Furthermore, MAK683 is currently being investigated in adults with advanced malignancies (NCT02900651 - ClinicalTrials.gov, 2024f). Similarly, DS-3201 (Valemetostat) is currently being investigated together with ipilimumab for the treatment of patients with metastatic prostate, urothelial and renal cell cancers (NCT04388852 - ClinicalTrials.gov, 2024a) and in patients with T-cell lymphoma (NCT04703192 - ClinicalTrials.gov, 2024o).

From all the known PKMTs, the inhibitors developed against EZH2 are arguably the most investigated at a clinical level (Chen et al., 2024; Duan et al., 2020). However, most inhibitors tested at clinical level focusing mostly on inhibiting tumour growth, with combination of said inhibitors with other therapeutics to inhibit tumour growth also gaining attention at a clinical level. However, whether such inhibitors also have potential in reversing chemoresistance has been given less importance at a clinical level. Thus, apart from the emphasis given to test the significance of these drugs at a preclinical level as evident from the information provided above or summarised in Table 5, further work is needed at a clinical level.

### 4.2 Targeting EHMT2/G9a

Several small-molecule G9a inhibitors have been investigated for their anti-cancer effects and have been used to enhance the sensitivity of cancer cells to traditional chemotherapy treatments. For instance, UNC0638 is a substrate-competitive, quinazoline-derivative G9A inhibitor that has been shown to reduce cancer cell viability, suppress migration and invasion and trigger apoptosis in neuroblastoma, breast and kidney cancer cells (Bellamy et al., 2020; Li R. et al., 2021; Liu et al., 2018). Furthermore, UNCO638 was found to induce apoptosis in EGFR-TKI resistant cells and its combination with erlotinib reduced tumour growth in NSCLC xenograft models (Wang L. et al., 2018). G9a inhibition promoted the re-expression of the PTEN gene, attenuating AKT signalling and reversing TKI resistance (Wang L. et al., 2018). Comparably, UNCO638 was found to enhance the sensitivity of resistant pancreatic cancer cells to gemcitabine by reducing the production of IL-8, which is implicated in stem-like properties and drug resistance (Pan et al., 2016). Indeed, the combination of UNC0638 and gemcitabine decreased cancer growth and metastatic properties of the gemcitabine-resistant cancer cells (Pan et al., 2016). In breast cancer, the combination of UNC0638 and the HDAC inhibitor CI-994 also induced apoptosis, decreased cancer stemness and reversed drug resistance (Lin et al., 2022).

UNC0642 is another substrate-competitive, quinazoline-derivate G9a small molecule inhibitor with better *in vivo* pharmacokinetic properties than UNC0638 and minimal cytotoxicity (Casciello et al., 2015). Its anticancer effects were shown in melanoma, bladder, breast and lung cancers (Cao et al., 2019; Dang et al., 2020; Pangeni et al., 2020; Yaqub et al., 2022). Moreover, G9a disruption through UNC0642 sensitised ovarian cancer cells to the PARP inhibitor Olaparib by disrupting DNA repair pathways and promoting DNA damage (Watson et al., 2019). Pangeni et al. (2020) also demonstrated that UNC0642 repressed the expression of CSC markers in lung cancer. Similarly, Zhou M et al., 2021 showed that UNC0642 reduced the survival and self-renewal capacity of leukemia stem cells, which are responsible for TKI resistance in CML.

Other G9a small molecule inhibitors like CM-272 and EML-741 that have demonstrated anti-tumoural and anti-neoplastic effects in different cancer sub-types, however more studies need to be carried out to investigate their effects on chemoresistance (Feoli et al., 2022).

### 4.3 Targeting other PKMTs

Although the majority of PKMT inhibitors are directed against EZH2 and G9a, other small molecules have been designed to target SYMD2, SYMD3, SETD7, SETD8 and SETDB1, with some found to aid in chemosensitivity.

Some SYMD2 inhibitors include BAY-598, AZ505, A893, EPZ033294 and LLY-507 (Fabini et al., 2019). In one study, BAY-598 reversed cisplatin resistance in NSCLC as it significantly decreased cell migration, tumour sphere number and induced apoptosis of cisplatin-treated lung cancer cells through the upregulation of p53 target genes including cyclin-dependent kinase inhibitor 1 (p21), Bcl-2-associated protein x (Bax) and Growth arrest and DNA damage-inducible protein GADD45 (GADD45) (Shang and Wei, 2019). The combination of AZ505,another a substrate-competitive SYMD2 inhibitor, together with cisplatin significantly inhibited tumour growth in NSCLC xenograft models when compared to AZ505 and cisplatin monotherapy groups (Shang and Wei, 2019).

SYMD3 is also a druggable target in cancer. For instance, BCI-121 is a substrate-competitive SYMD3 inhibitor that was found to impair the cell viability of medulloblastoma, CRC and ovarian cancer cells (Asuthkar et al., 2022; Jiang et al., 2019; Peserico et al., 2015). BCI-121 also downregulated cyclin D1/D3 and upregulated retinoblastoma (Rb) expression in medulloblastoma patient-derived xenografts, however at a high concentration BCI-121 was found to be significantly cytotoxic in the primary tumour samples (Asuthkar et al., 2022). Additionally, BCI-121 produced a dose-dependent inhibition of adhesion and invasion in ovarian cancer spheroids (Lyu et al., 2020). Nonetheless, more studies are required to investigate its effect with other chemotherapeutics and its benefit in reversing drug resistance. EPZ031686, diperodon and MS2177 are different SYMD3 inhibitors that also require further investigation as anti-cancer therapeutics.

(R)-PFI-2 is a SETD7 inhibitor that has been shown to increase the sensitivity of NSCLC cells to doxorubicin (Daks et al., 2021). Cyproheptadine is an antagonist of the histamine and serotonin receptor but also a SETD7 inhibitor. It was found to induce apoptosis in multiple myeloma cells by inhibiting PI3K/AKT signalling, a key signalling cascade involved in chemoresistance (Li et al., 2013). Hence, cyproheptadine could potentially aid in the reversal of SETD7-induced chemoresistance, however further investigations are required.

No specific small molecules directed towards SETDB1 have yet been characterised. However, studies have shown that this PKMT can be targeted through non-specific compounds such as chaetocin, paclitaxel, mithramycin and 3-deazaneplanocin A (DZNep) (Karanth et al., 2017; Liao et al., 2023). All have shown positive therapeutic effects in cancer cell lines, as reviewed by Karanth et al. (2017). For example, Hou et al. (2020) showed that mithramycin enhanced cetuximab sensitivity in KRAS-mutated CRC cells. The combination of cetuximab and mithramycin significantly reduced the size of CRC tumour xenografts as compared to either of the therapies administered individually. Further, treatment with this combination also attenuated PI3K/AKT signalling in the KRAS-mutated CRC cells (Hou et al., 2020).

UNC0379 is a substrate-competitive SETD8 inhibitor that has shown synergistic effects with the alkylating agent, melphalan in multiple myeloma (Herviou et al., 2021). In addition, UNC0379 suppressed the invasion and migration abilities of pancreatic cancer cells and decreased the proliferation of ovarian cancer cells through the induction of apoptosis (Liu M. et al., 2021; Wada et al., 2020).

Lastly, Zhang and his colleagues (2024a) have recently reviewed other PKMT inhibitors and their contribution to reversing chemoresistance in lung cancer. The inhibitors discussed in relation to resensitising lung cancer cells target either EZH2 (GSK126, GSK343, DZNep, GSK343, EPZ011989), G9a/EHMT2 (UNC0642, UNC0638), DOT1L (SGC0946), SMYD3 (EPZ031686) or SMYD2 (BAY-598), some of which were also discussed in this review.

### 4.4 Targeting type I PRMTs

Type I PRMT inhibitors are typically not designed to be selective towards one particular PRMT. For instance, MS023 is a potent substrate binding-site inhibitor of type I PRMTs (Eram et al., 2016). It has shown anti-tumour effects in breast cancer cells by activating an antiviral response characterised by increased interferon expression and double-stranded RNA (dsRNA) accumulation, which induces cell death (Wu et al., 2022). Treatment with MS023 in HT29 colon cancer cells significantly downregulated pathways involved in migration and invasion and increased the expression of adhesion proteins (Plotnikov et al., 2020). MS023 also reduced the clonogenic growth of ovarian cancer cells exposed to cisplatin, sensitising them to apoptosis (Musiani et al., 2020). MS023 was also found to upregulate genes involved in mitosis and cell-cycle regulation inducing apoptosis in Srsf2^P95H^ mutant AML preferentially to wild-type cells (Fong et al., 2019). As for the use/role of MS023 in chemoresistance, its potential use is still poorly understood, however when used in with combination Talazoparib (PARP inhibitor - PARPi), it helped resensitise PARPi resistant ovarian cells to treatment, thus suggesting that type I PRMT inhibitors could mitigate resistance to PARPi inhibitors (Dominici et al., 2021).

GSK3368715 is a SAM uncompetitive type I PRMT inhibitor that entered the clinical trials (NCT03666988 - ClinicalTrials.gov, 2022a) for the treatment of solid tumours and diffuse large B-cell lymphoma. Unfortunately, it was terminated as the benefit/risk profile did not favour continuation of the study. Nonetheless, recently this inhibitor has shown to increase gemcitabine sensitivity in human and murine PDAC and PDAC PDXs, as well as delay the development of gemcitabine resistance in human PDAC xenograft models (Nguyen et al., 2024). Thus, there is still potential that this inhibitory may be of benefit in future clinical studies, particularly for reversing or preventing chemoresistance.

DB75 and TC-E5003 are two selective PRTM1 inhibitors which have been reported to reverse gemcitabine resistance in PDAC at pre-clinical level, by inhibiting the HSP70–BCL2 pathway (Wang et al., 2020a). However, their potential at a clinical level has not been tested yet, and their exact mode of inhibition is also not fully understood.

SGC707 is an allosteric PRMT3 inhibitor (Kaniskan H.Ü et al., 2015). Treatment of pancreatic cancer cells with SGC707 significantly reduced the expression of ABCG2, which sensitised the cells to gemcitabine (Hsu et al., 2018). In addition, SGC707 has been shown to inhibit glycolysis and tumour growth in hepatocellular carcinoma and glioblastoma cells (Lei et al., 2022; Liao et al., 2022). In CRC cells, SGC707 inhibited Vascular Endothelial Growth Factor A (VEGFA) expression and significantly reduced their migration and invasion abilities (Zhang et al., 2021).

### 4.5 Targeting PRMT5

Many PRMT5 inhibitors have shown promising pre-clinical efficacy, with some now being investigated in clinical trials. The review by Feustel and Falchook (2022) discusses the recent PRMT5 inhibitors that have made it to oncology clinical trials. One is JNJ-64619178, an inhibitor of the PRMT5-methylosome protein 50 (MEP50) complex that binds the SAM- and substrate-binding pockets (Brehmer et al., 2021). It is currently being studied in phase I clinical trials (NCT03573310 - ClinicalTrials.gov, 2024h) as a monotherapy in patients with advanced solid tumours and B-cell non-Hodgkin lymphoma (Vieito et al., 2023). Additionally, JNJ-64619178 was investigated as a synergistic tool to enhance the efficacy of Trametinib (MEK1/2 kinase inhibitor) in glioblastoma. The combination of both drugs showed an increase in the number of apoptotic cancer cells, caspase 3/7 activity and the number of cells in G1 cell cycle arrest than either individual therapy alone (Banasavadi-Siddegowda et al., 2022). Likewise, the combination of the PRMT5 inhibitor and LB100 (a protein phosphatase 2A (PP2A) inhibitor) enhanced G1 cell cycle arrest in glioblastoma cells (Otani et al., 2021).

GSK3326595 is a non-SAM competitive and substrate-competitive PRMT5 inhibitor. In mantle cell lymphoma cell lines, treatment with GSK3326595 resulted in a loss of viability and increased DNA damage characterised by downregulation of defects in DNA-damage response (DDR) genes, increased reactive oxygen species (ROS) and gamma-H2AX foci (Che et al., 2023). GSK3326595 has been studied in patients with solid tumors and non-Hodgkin’s lymphoma (NCT02783300 - ClinicalTrials.gov, 2023a), where it demonstrated positive clinical activity and manageable side effects (Siu et al., 2019). It has also been investigated in early-stage breast cancer (NCT04676516 - ClinicalTrials.gov, 2022c), but no results have been published yet for this study.

The inhibitor EPZ015666 targets the peptide-binding site of PRMT5. It has shown significant anti-tumour activities in retinoblastoma, multiple myeloma, breast, bladder and cervical cancers (Liu X. et al., 2021; Gao et al., 2021; Gullà et al., 2018; Hu et al., 2018; Vinet et al., 2019). In glioblastoma, EPZ015666 overcame therapeutic resistance to the mTOR inhibitor PP242 via synergistic tumour inhibitory effects *in vitro* and xenograft mouse models in combination with the mTOR inhibitor PP242 (Holmes et al., 2019). Glioblastoma cells treated with a combination of EPZ015666 and PP242 resulted in a significant loss of cell viability, an increase in the number of apoptotic cells and a reduction in the expression of c-MYC and cyclin D1 (Holmes et al., 2019). Similarly, in xenograft models, the combinatorial treatment was markedly more effective in terms of tumour growth inhibition than either monotherapy (Holmes et al., 2019).

GSK3203591 (a.k.a GSK591) is another inhibitor of PRMT5-MEP50 activity. It has been shown to induce cancer apoptosis and promote chemosensitivity to resveratrol in lung cancer cells by downregulating the AKT/GSK3β/cyclin D and E signalling pathway (Li Y. et al., 2019). In agreement, Zhang et al. (2019) showed that GSK591 suppressed lung cancer cell proliferation by downregulating phosphorylated AKT together with cyclins D and E, preventing cell cycle progression. Moreover, GSK591 significantly reduced the proliferation and self-renewal potential of breast CSCs, an effect that was enhanced in combination with 4-hydroxytamoxifen (Chiang et al., 2017). It was also shown to be 10-fold more effective at killing Srsf2^P95H^ mutant AML compared to the WT counterpart (Fong et al., 2019).

PRT543 is a potent, selective, oral PRMT5 inhibitor that binds to the substrate recognition site of PRMT5 and inhibits its methyltransferase activity. It was shown to have pre-clinical antitumour activity in Splicing Mutant Myelodysplastic Syndrome (MDS) and Acute Myeloid Leukemia (AML) (Schwartz et al., 2021). Also at pre-clinical stage, PRT543 on Splicing factor 3b subunit 1 (SF3B1) uveal melanoma MEL202 (SF3B1^R625G^ active mutant) and MEL270 (SF3B1^WT^) cell lines showed downregulation of SF3B1 target genes associated with increased intron retention, predominantly in the MEL202 mutant. In combination with DNA-alkylating agents or PARP inhibitors, PRT543 produced a synergistic reduction in cell viability through the regulation of cancer-associated RNA splicing machinery and the DNA damage response (Ito et al., 2021). This led to the open-label phase 1 study NCT03886831 (ClinicalTrials.gov, 2023b), which assessed the safety and efficacy of PRT543 in 23 unselected patients with refractory disease to established therapies, 12 with myelofibrosis and 11 with myelodysplastic syndrome. The results showed that PRT543 was safe and tolerated, presenting a dose-dependent inhibition of PRMT5. It is thus being evaluated either as monotherapy in patients with at least one spliceosome mutation or in combination with ruxolitinib in myelofibrosis patients demonstrating a sub-optimal response to ruxolitinib (Patel et al., 2021). The safety, tolerability and preliminary efficacy of PRT543 was also analysed in another cohort consisting of 56 patients with recurrent and/or metastatic adenoid cystic carcinoma (ACC), since there is currently no approved systemic treatment. The results showed that PRT543 was tolerable and 57% of the patients benefited from PRT543 administration, with 70% of patients presenting stable disease, however the efficacy was limited (Ferrarotto et al., 2024). At pre-clinical level, PRT543 has shown that when used in combination with venetoclax (a BCL-2 inhibitor), it sensitised a mouse model of ibrutinib-resistant mantle cell lymphoma (MCL). Combination treatment with well-tolerated doses of venetoclax and PRT543 in MCL *in vivo* models showed synergistic anti-tumour activity without evidence of toxicity, thus justifying that this combination strategy could be advanced to clinical setting (Brown et al., 2019).

The recent inhibitor, AZD3470, which inhibits PRMT5 activity by binding to the PRMT in the presence of methylthioadenosine (MTA), in methylthioadenosine phosphorylase (*MTAP*)-deficient tumor cells (Spira et al., 2024), has recently been developed by AstraZeneca and is currently being tested in two different clinical trials (NCT06137144 - ClinicalTrials.gov, 2024p and NCT06130553 - ClinicalTrials.gov, 2024g). In one of the clinical trials undergoing (NCT06137144), this inhibitor is being tested as a monotherapy in combination with other anticancer agents (not provided) in patients with relapsed/refractory haematologic malignancies. Among the participants anticipated in this study, patients who have previously received at least three prior lines of therapy for the treatment of Classical Hodgkin lymphoma (cHL), and must have exhausted all available therapies with demonstrated clinical benefit are expected to take part. Through this study, the safety, tolerability, pharmacokinetics and preliminary efficacy following oral administration of AZD3470 as monotherapy or in combination will be investigated. Thus, this novel PRMT5 inhibitor can serve as potential therapy to sensitise patient to therapy used for cHL.

The inhibitor DS-47, which targets not only PRMT5, but also PRMT7, is a SAM-competitive inhibitor which has been tested against PRMT5 inhibition. Pharmacological inhibition of PRMT5 by DS-437 resulted in reduced human regulatory T cells functions and inhibited the methylation of FOXP3 at R51. Furthermore, this inhibition resulted in enhanced anti-tumour effects of anti-erbB2/neu monoclonal antibody targeted therapy in Balb/c mice having CT26Her2 tumours by inhibiting regulatory T cells function and induction of tumour immunity (Nagai et al., 2019). Moreover, the same inhibitor also demonstrated that inhibition of PRMT5 significantly reduced gemcitabine resistance in pancreatic cancer caused by UBR7 depletion (Table 4) (Bi et al., 2023). Despite presenting potential for reversing resistance, this inhibitor is yet to be investigated at a clinical level. Lastly, there are other PRMT5 inhibitors which have been tested at pre-clinical level, including some for reversing chemoresistance (PRT382, YQ36286), as well reviewed by others (Feustel and Falchook, 2022).

## 5 Conclusion and future directions

Drug resistance is one of the major challenges of cancer treatment since it greatly limits the therapeutic options for the patient and worsens the prognosis. With the current push towards more personalised medicine, combining current chemotherapeutic regimens with specific PMT inhibitors could in the future be a strategy to ensure that a sub-set of patients can successfully benefit in the long-term from the treatment being administered. However, in order to achieve this there is a need for better understanding of the role of PKMTs and PRMTs in chemoresistance, through the collection of more comprehensive data from well-characterised patient cohorts. This is because most of the current published work on PMT inhibitors has focused on inhibiting enzymes which are dysregulated in relation to tumorigenesis and not chemoresistance, such that a more focused investigation is required in relation to whether chemoresistance can be reversed when administering such inhibitors and the identification of chemoresistance-specific targets.

Despite being discovered years ago (Ambler and Rees, 1959), protein methylation has not been as widely studied as other PTMs due to the lack of confident identification, efficient enrichment strategies and experimental techniques (Deng et al., 2016; Levy, 2019; Micallef and Baron, 2023; Wang et al., 2017). In particular there needs to be a thorough understanding of the biological significance and role of non-histone proteins as substrates of PMTs in chemoresistance, which is still far from properly characterised. Technological advances in mass spectrometry for PTM identification as well as various methodological developments in the analysis of methyltransferase expression and activity will be required to obtain crucial information from clinical samples.

Another important aspect to investigate is how PMTs are involved in the mechanism of the different chemotherapeutic drugs and lead to cancer cell evasion once dysregulated, which also requires the collection of much more evidence from different sources. The dysregulation of both PKMTs (such as EZH2, G9A, SETD7, SETD8, SYMD2 and SYMD3) and PRMTs (such as PRMT1, PRMT3, PRMT5 and PRMT6) in tumours has been shown to promote drug resistance in various ways such as by CSC renewal, activation of key signalling pathways like the Wnt/Notch pathway and through cell cycle progression. This would provide key mechanistic information and insight into how to design future inhibitors which would be specifically synergistic with selected chemotherapeutic drugs. Targeting PMTs strategically using small inhibitory molecules would aid in the reversal of drug resistance, thus promoting cell death within the evading cancer cells.

Currently inhibitors are only available against a very small number of the known PMTs and the specificity and sensitivity of the available molecules are not always of the desired quality. In order to improve their efficacy, apart from developing more iterations of these inhibitory molecules, it would be ideal to design new inhibitory mechanisms. However, this option is somewhat limited considering that all PMTs use the same donor and mechanism to add methylation, and they fall within 2 families making their active sites particularly similar. Recently, the use of proteolysis-targeting chimera (PROTAC) or enzyme-substrate adaptor interaction inhibitors to inhibit methylation events mediate by PMTs have started to be implement against some PKMTs (Velez et al., 2023; Velez et al., 2024) and PRMTs (Martin et al., 2024; McKinney et al., 2021; Shen et al., 2020). These two approaches could be implemented to the development of future inhibitors against PMTs, offering additional novel inhibitory mechanisms to the ones already implemented (Wang M. Y. et al., 2022). Nevertheless, irrespective of the route chosen, therapeutic targeting of PKMTs and PRMTs is expected to continue expanding in the near future.

However, to do this effectively there needs to be a clear understanding of why a number of these inhibitors have failed in either pre-clinical or clinical trials. PMT inhibitors have failed to demonstrate sufficient therapeutic efficacy at non-toxic doses in clinical trials. Their broad range of targets results in unacceptable side effects that outweigh their potential benefits. This effectively translates into a very narrow therapeutic range for an effective dose that has acceptable toxicity in humans, making achieving therapeutic effects safely extremely challenging. Building on this, more clinically relevant inhibitor testing and better biochemical understanding will also allow for an improvement in *silico* prediction and assist in shortlisting further potential PMT inhibitors. Moreover, we still lack many specifics on the mode of action of each inhibitor, hindering the design of better inhibitors. The similarity between the active sites of enzymes within the same family make PMT inhibitor specificity probably the greatest challenge to overcome in PMT inhibitor targeting for safe clinical application. Considering that each enzyme has hundreds of substrate proteins fulfilling very diverse cellular roles it is expected that perturbing the enzyme concentration will have implications on numerous biochemical pathways, some of which may be essential and non-redundant for non-cancerous cells.

A final point to consider is that the ability of cancer cells to alter the activity of specific proteins through PTMs including methylation is only one of the mechanisms by which tumours develop chemoresistance. Thus it might be necessary to use multiple inhibitory molecules in combination in order to reverse chemoresistance. Further investigating the significance of combinatory targeting with other, already approved, drugs such as immunomodulatory (particularly immune checkpoint inhibitors) or signalling pathway modulatory molecules (PI3K, MAPK or Wnt pathway inhibitors) along with PMT inhibitors might be one way of going about it. Finding the right balance between the different inhibitors will be extremely challenging, given the key role such pathways play, but a potential avenue nonetheless. Moreover, a similar issue which will have to be faced has to do with the development of resistance against the PMT inhibitors themselves, something which has already started being observed for some PMTs, such as the EZH2 inhibitors (Chen et al., 2024; Duan et al., 2020; Feoli et al., 2022). Thus, solving one issue can result in another problem arising and so forth.
